# Supplementary material for: Action initiation and punishment learning differ from childhood to adolescence while reward learning remains stable
Source: Nat Commun. 2023 Sep 14;14:5689. doi: 10.1038/s41467-023-41124-w (PMC10502052; doi:10.1038/s41467-023-41124-w)
Supplement: Supplementary file 1 — Supplementary Information New [file 41467_2023_41124_MOESM1_ESM.pdf]

Supplementary Information for ‘Action initiation and punishment learning differ from childhood to adolescence while reward learning remains stable’

Ruth Pauli, Inti Brazil, Gregor Kohls, Miriam C. Klein-Flügge, Jack C. Rogers, Dimitris Dikeos, Roberta Dochnal, Graeme Fairchild, Aranzazu Fernández-Rivas, Beate Herpertz-Dahlmann, Amaia Hervas, Kerstin Konrad, Arne Popma, Christina Stadler, Christine M. Freitag, Stephane A. De Brito, Patricia L. Lockwood

Correspondence should be addressed to:
Ruth Pauli, Centre for Human Brain Health, University of Birmingham, United Kingdom. E-mail: r.pauli@bham.ac.uk

Patricia L. Lockwood, Centre for Human Brain Health, University of Birmingham, United Kingdom. E-mail: p.l.lockwood@bham.ac.uk

This PDF file includes:

Supplementary Notes
Figures S1 to S16
Tables S1 to S4

Supplementary Notes

Behavioural responses by stimulus point value

We plotted participants' behavioural responses over stimuli repetitions, by the point value of the stimuli (see Figure S1). Punishment stimuli clustered together, as did higher value reward stimuli. This suggests that participants' subjective response to the point values was non-linear, which is commonly observed in the literature (e.g.,[1–3]). However, we note that with only four stimuli each for reward and punishment trials, it would be premature to draw conclusions about the effect of point value on participants' behaviour. In addition, our computational models suggested that magnitude information was not a strong driver of participants' behaviour.

Behavioural responses by three pubertal stages

As an additional control analysis, we collapsed the pubertal status scores into three categories (pre/early, mid, and late/post pubertal) to reflect the three age categories used for visualisation. Collapsing the pubertal stage data in this way did not change our results. Consistent with the analysis using the original five stages of the PDS measure, we still observed main effects of puberty on the number of correct responses (OR = 1.06 [1.03, 1.11], $z = 3.72$, $p < .001$), a puberty by repetition interaction (OR = 1.02 [1.01, 1.04], $z = 2.60$, $p = .009$, and a puberty by valence interaction (OR = 1.17 [1.13, 1.22], $z = 6.48$, $p < .001$), but no puberty by repetition by valence interaction (OR = 1.02 [0.98, 1.05], $z = 0.89$, $p = .37$).

Associations with age are similar for model 6 parameters

We confirmed that our main results were unchanged if we selected model 6 (with the additional single magnitude sensitivity parameter) rather than model 5 as the winning model. As before, age was strongly associated with increased punishment learning rates ($\beta = 0.10$ [0.05, 0.15], $z = 4.26$, $p < .001$), and lower action initiation biases ($\beta = -0.20$ [–0.28, –0.12], $z = -4.78$, $p < .001$; see Figure 4). Reward learning rates did not differ significantly with age ($\beta = 0.01$ [–0.06, 0.07], $z = 0.17$, $p = .86$). We observed a weaker negative relationship between magnitude sensitivity and age ($\beta = -0.09$ [–0.17, –0.01], $z = -2.26$, $p = .02$), and no relationship between age and temperature parameter ($\beta = 0.002$ [–0.07, 0.08], $z = 0.06$, $p = 0.95$).

We also tested for quadratic effects of age by adding age^2 terms to the models. However, none of the model parameters exhibited significant quadratic associations with age (temperature parameter: β = 0.99 [–0.87, 2.85], z = 1.04, p = .30. Reward learning rate: β = -0.83 [–2.37, 0.70], z = –1.07, p = .29. Punishment learning rate: β = 0.37 [–0.80, 1.54], z = 0.62, p = .54. Action initiation bias: β = –0.57 [–2.63, 1.49], z = –0.54, p = 0.59. Magnitude sensitivity: β = –0.10 [–2.10, 1.90], z = –0.10, p = .92).

We re-ran the same models with pubertal stage rather than chronological age. These analyses revealed a similar positive association with punishment learning rate (β = 1.41×10^{-4} [6.20×10^{-5}, 2.20×10^{-4}], z = 3.48, p <.001), a negative association with action initiation bias (β = –0.001 [2.00×10^{-3}, 5.00×10^{-4}], z = –3.40, p = .001), and no significant association with reward learning rate (β = 5.4×10^{-5} [-5×10^{-5}, 1.6×10^{-4}], z = 1.02, p = .31). There was also a negative association with magnitude sensitivity (β = –0.01 [–0.01, –0.001], z = –2.21, p = .03) and no significant association with temperature parameter (β = -2×10^{-6} [-5×10^{-6} 10×10^{-6}], z = –1.29, p = .20).

Correlations between model parameters and task performance

Correlations between model parameters and task performance (proportion of correct responses) for reward and punishment stimuli are shown in Table S3. All parameters exhibited at least one significant correlation, indicating that all our model parameters meaningfully captured elements of performance.

Reanalysis with all 832 eligible participants and no exclusion of outliers

To confirm that our results were not driven by the exclusion of poor-quality data or outliers on model parameters, we repeated our main analyses using the data from all 832 eligible participants (i.e., including those who never responded (n = 4), responded on every trial (n = 2), scored below zero points (n = 6), or responded on fewer than half of the reward trials (n = 78). Analysis of model parameter and age associations were repeated both with and without outliers, i.e., model parameter values more than three standard deviations from the mean. These analyses revealed virtually identical findings to our main analyses (see Table S2 and Figure S12).

We also note that there was no significant age difference between the excluded participants and those included in the final sample (2-tailed t-test: $t_{(110.94)} = 0.25$, $p = .81$, $BF_{01} = 7.87$, moderate evidence for the null).

Reaction time across stimulus repetitions by age

We analysed whether reaction times for 'go' responses across the task were related to age. A linear mixed effects model predicting reaction time from stimulus repetition, sex, and IQ (fixed effects) and participant nested in site of data collection (random effects) revealed a significant main effect of age on reaction times, with older participants reacting faster ($\beta = -23.37$ [−40.55, −6.18], $t_{(40084)} = -2.67$, $p = .008$). There was also a main effect of stimulus repetition on reaction times, with faster reaction times for later repetitions ($\beta = -53.67$ [−58.62, −48.73], $t_{(40084)} = -21.29$, $p < .001$), and a significant age by repetition interaction ($\beta = -9.69$ [−14.63, −4.75], $t_{(40084)} = -3.85$, $p < .001$), with reaction times decreasing more rapidly for older participants (see Figure S13). The age-related patterns in reaction times are similar to previous learning experiments where reaction times decrease when actions are learned, and fit with previous developmental studies suggesting age related reaction time decreases in general[4,5].

Declining action initiation bias model

As a control analysis we considered an additional model that accounted for the action initiation bias as declining over time, rather than a constant action initiation bias as in our winning model. The amount of decline with time was set as a linear free parameter. The winning model still outperformed the declining action initiation bias model (exceedance probability = 0.99, $\Delta BIC_{int} = -1371.82$) so we did not consider the additional model further.

Supplementary Figures

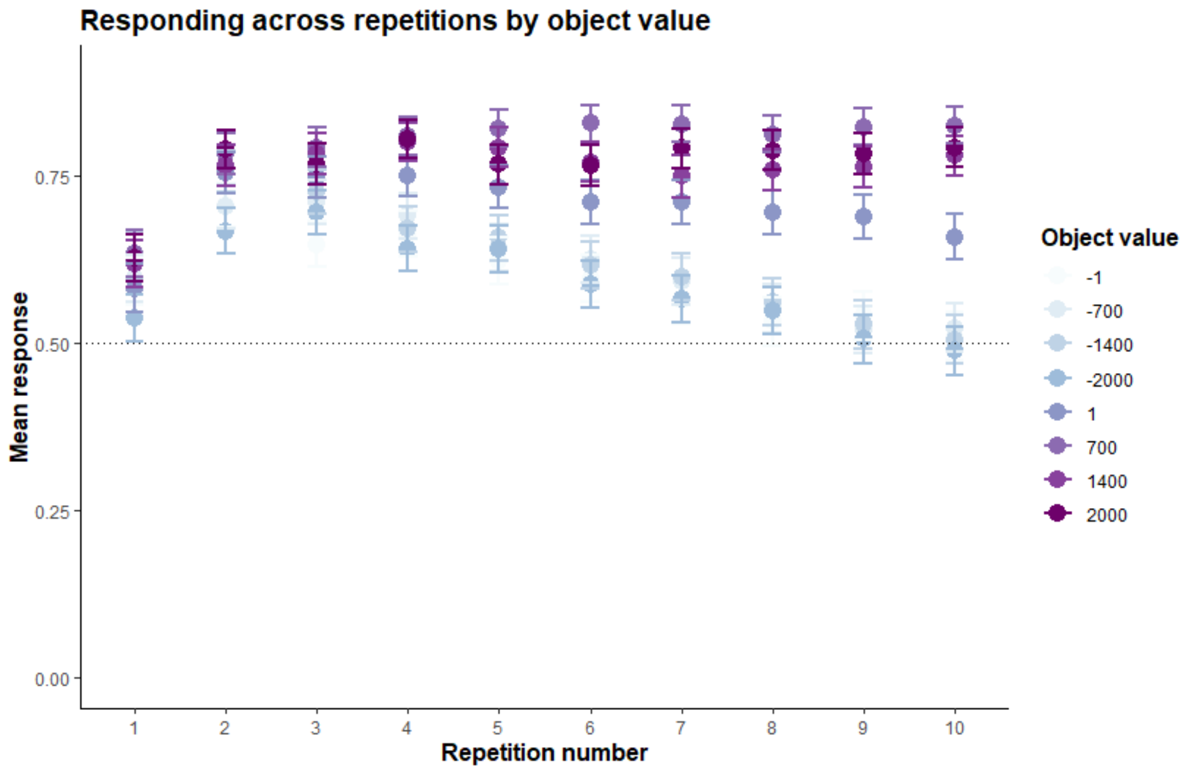

Figure S1. Responding across repetitions by object value. Responses to stimuli across repetitions, according to object point value. Responses to reward objects (with values of 1, 700, 1400, or 2000) remain fairly similar after the second presentation, with the exception of the lowest value reward object (1), where responses decrease again towards the end of the task. Responses to all punishment objects decrease steadily after the second presentation. Points and error bars represent means and 95% confidence intervals of the means.

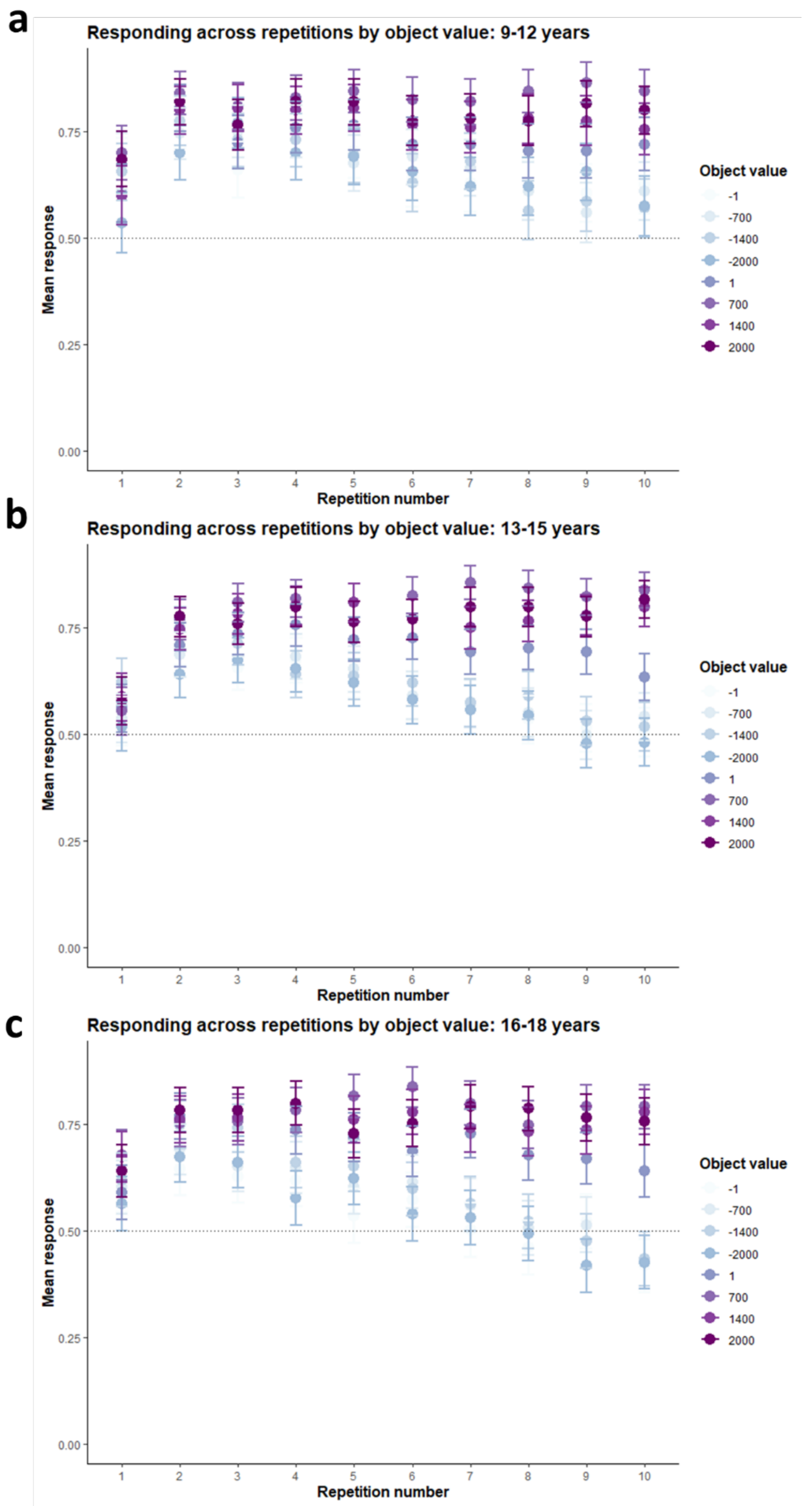

Figure S2. Responding across repetitions by object value for three age groups. (a) Responses by participants aged 9-12 years (N = 200). **(b)** Responses by participants aged 13-15 years (N = 303). **(c)** Responses by participants aged 16-18 years (N = 239). The pattern of responding to all reward magnitudes was broadly similar across all three age groups, while older participants showed decreased responding to all punishment magnitudes compared to younger participants. Points and error bars represent means and 95% confidence intervals of the means.

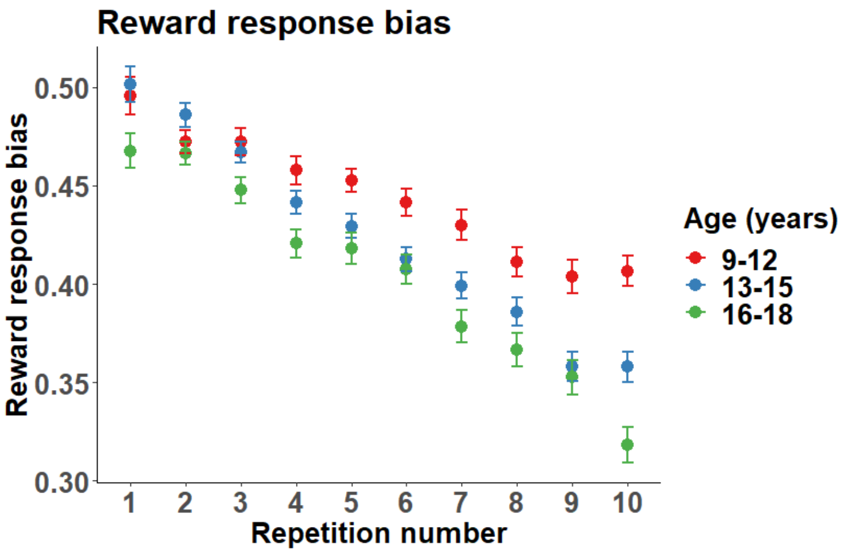

Figure S3. Reward response bias across stimulus repetitions by age. There was a significant negative association between age and overall reward response bias (GLMM: β = –0.12 [–0.16, –0.08], $t_{(58352)}$ = –6.31, p < .001, 2-sided) as well as a significant age by repetition interaction (β = –0.05 [–0.06, –0.05], $t_{(58352)}$ = –15.84, p < .001, 2-sided). Points and error bars represent means and 95% confidence intervals of the means. Age was continuous in the analysis; age bins are for presentation purposes only. N = 742.

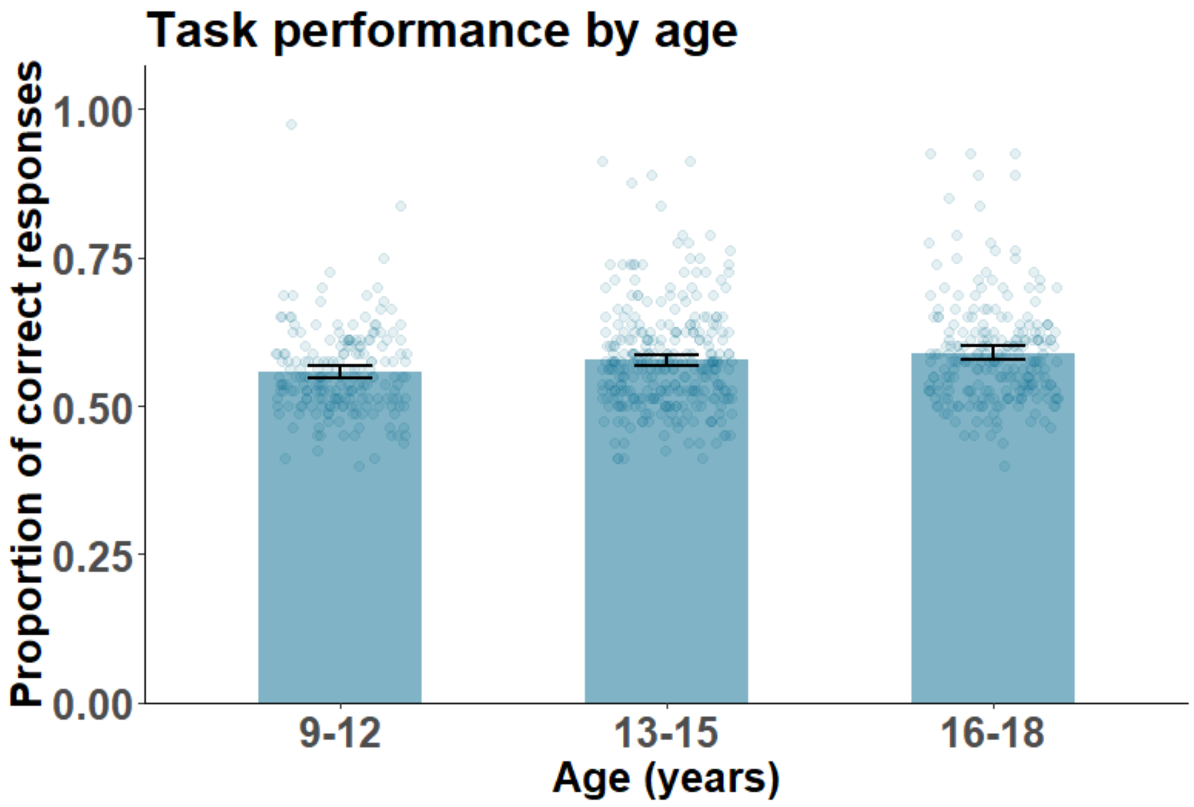

Figure S4. Overall task performance by three age groups. Performance improved with age (GLMM: OR = 1.08 [1.04, 1.11], z = 4.58, p <.001, 2-sided). Error bars

represent 95% confidence intervals of the mean. Age was continuous in the analysis; age bins are for presentation purposes only. N = 199 aged 9-12, 302 aged 13-15, and 235 aged 16-18.

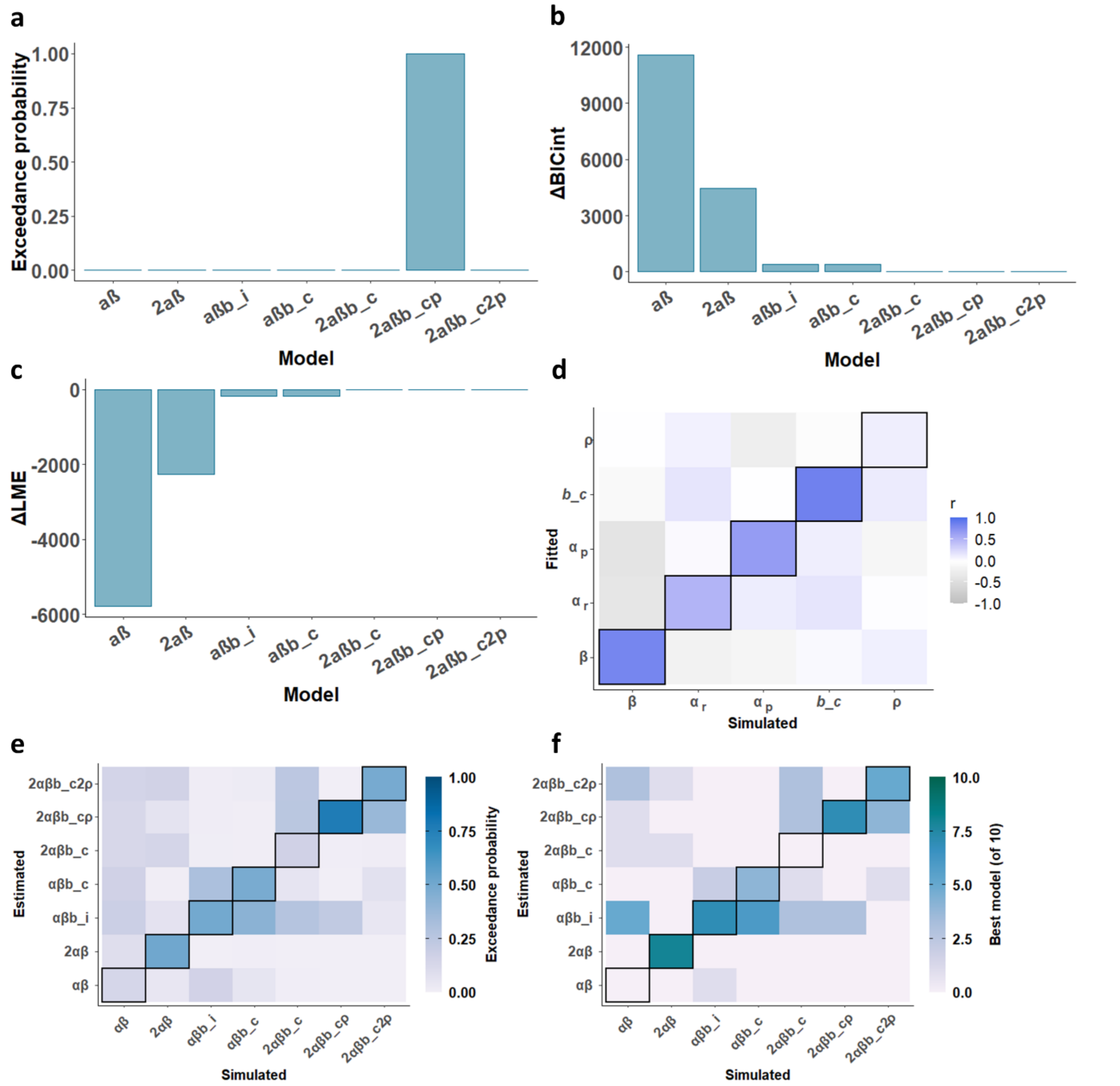

Figure S5. Model performance and validation for the initial seven models. (a) Exceedance probability for the seven computational models that comprised the model space. The winning model on this measure was the 2αβ*b_c*ρ model, with separate reward and punishment learning rates, a constant action initiation bias, and a magnitude sensitivity parameter. **(b)** ΔBIC_{int}, relative to the winning model (2αβ*b_c*). **(c)** ΔLME, relative to the winning model (2αβ*b_c*ρ). **(d)** Parameter recovery for model 6 (2αβ*b_c*ρ). The confusion matrix represents Spearman correlations between simulated and fitted (recovered) parameters. Each parameter exhibited a significant positive correlation between its true and fitted values, with *r* values ranging from 0.1 – 0.83 (shown on the lower diagonal). However, the recoverability for the magnitude

sensitivity parameter ρ was poor. **(e)** Exceedance probability from the model identifiability procedure. The diagonal represents the probability of each model having the best fit to its own synthetic data. **(f)** Number of runs where each model was selected as the best fit for data generated by each model in the model identifiability procedure. The diagonal represents the number of runs each model was selected as the best fit for its own data. Although model 6 (2αβ*b_c*ρ) won on two of the three performance measures (exceedance probability and ΔLME), the magnitude sensitivity parameter did not recover well, so we selected model 5 as the overall winning model. Model 5 also had the lowest absolute ΔBIC_{int}.

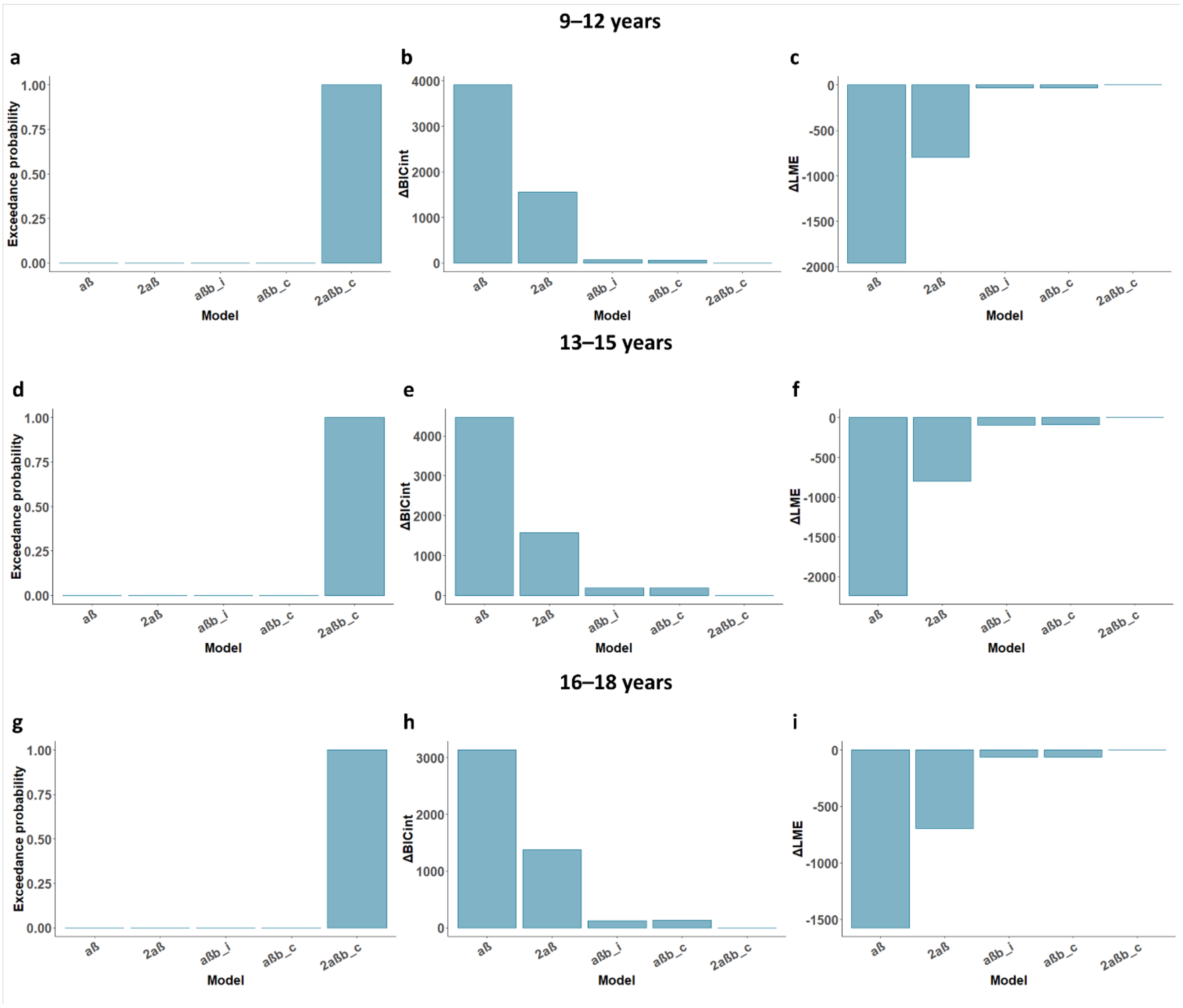

Figure S6. Model comparison for three separate age groups. The final step in the modelling procedure (i.e., comparison of all five models) was replicated in three separate age groups: 9–12 years, 13–15 years, and 16–18 years. **(a)** Exceedance probability for models with 9–12-year-old participants. **(b)** ΔBIC_{int} for models with 9–12-year-old participants. **(c)** ΔLME for models with 9–12-year-old participants. **(d)** Exceedance probability for models with 13–15-year-old participants. **(e)** ΔBIC_{int} for

models with 13–15-year-old participants. **(f)** ΔLME for models with 13–15-year-old participants. **(g)** Exceedance probability for models with 16–18-year-old participants. **(h)** ΔBIC_{int} for models with 16–18-year-old participants. **(i)** ΔLME for models with 16–18-year-old participants. In each age group, the winning model was the same as for the overall sample on all three measures of performance.

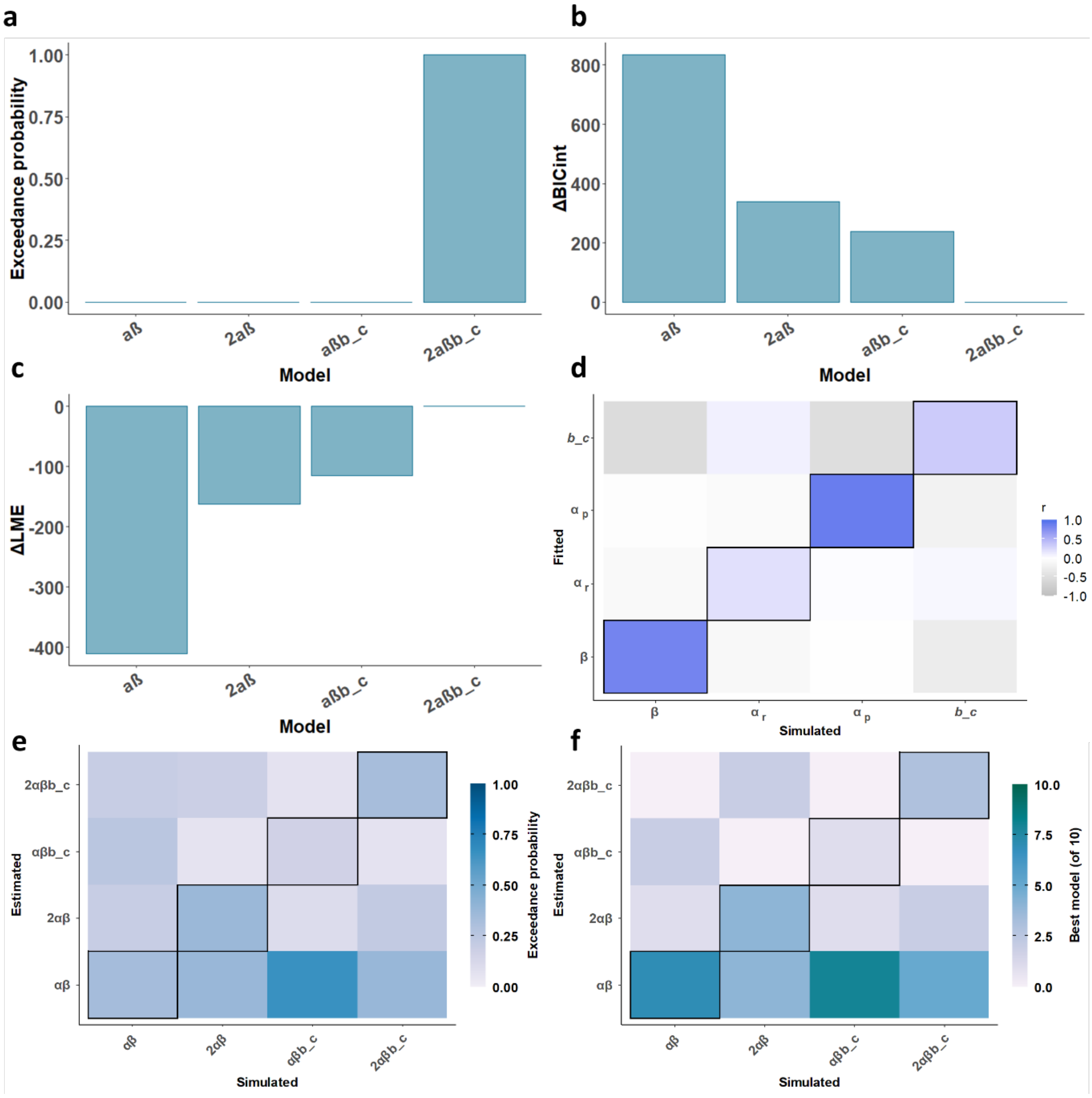

Figure S7. Model comparison with stimulus repetitions 2-10 only. (a) Exceedance probability for the four computational models that comprised the model space when including data from repetitions 2-10 only. The winning model on this measure was the same as when all stimulus repetitions were included (2αβ*b_c* model). **(b)** ΔBIC_{int}, relative to the winning model (2αβ*b_c*). **(c)** ΔLME, relative to the winning model (2αβ*b_c*). Note model 3 is not included in the model space since there can be no initial action initiation bias when omitting the first stimulus presentations. **(d)** Parameter recovery. The confusion matrix represents Spearman correlations between simulated and fitted (recovered) parameters. Parameters for the winning 2αβ*b_c* model were

recoverable. **(e)** Exceedance probability from the model identifiability procedure. The diagonal represents the probability of each model having the best fit to its own synthetic data. **(f)** Number of runs where each model was selected as the best fit for data generated by each model in the model identifiability procedure. The diagonal represents the number of runs in which each model was selected as the best fit for its own data. Model identifiability was reasonable although less strong than for the models using all 10 stimulus repetitions.

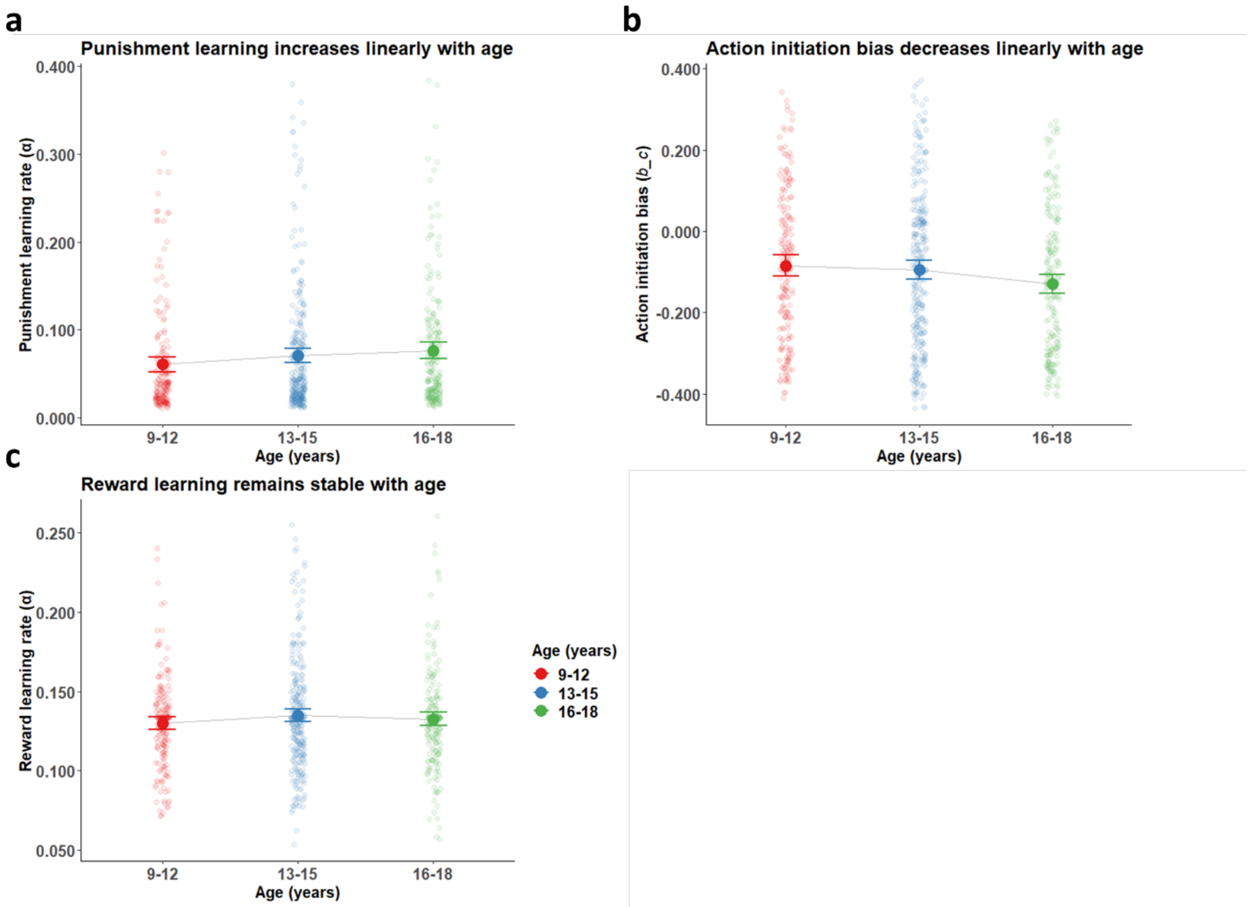

Figure S8. Age differences in action initiation bias, punishment learning, and reward learning from the winning model with stimulus 2-10 repetitions only. (a) Punishment learning rate across three age groups. Punishment learning rates increased linearly with age. **(b)** Action initiation bias across three age groups. Action initiation biases declined linearly with age. **(c)** Reward learning rates across three age groups. Reward learning rates remained stable with age. Points and error bars represent means and 95% confidence intervals of the means for each group, with raw data represented by smaller points. N = 199 aged 9-12, 302 aged 13-15, and 235 aged 16-18. Division into age groups is for presentation purposes only; age was treated as a continuous variable in all analyses. The relevant statistical analyses are provided in Table S2.

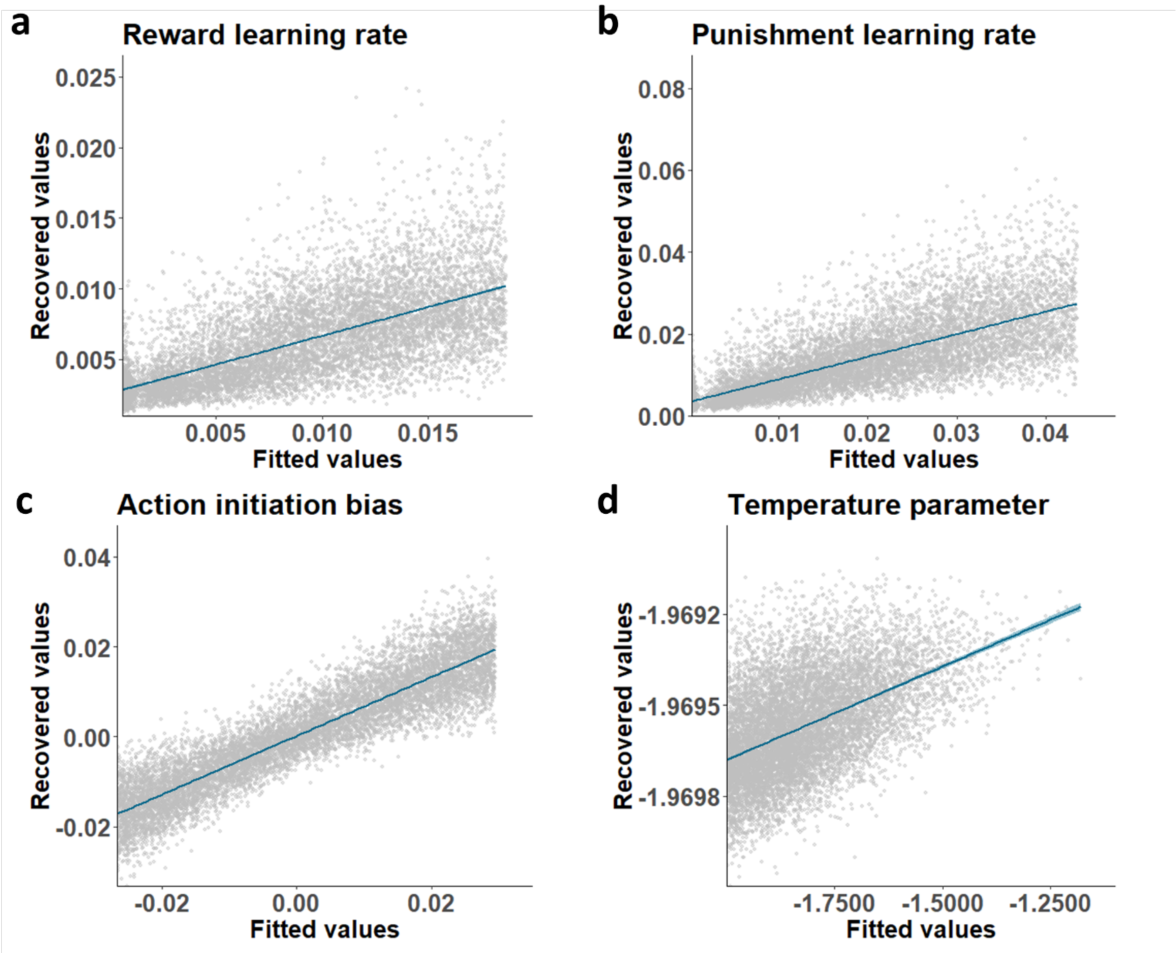

Figure S9. Spearman correlations between fitted and recovered values for model parameters from the winning model. All correlations were significant. **(a)** Correlation between fitted and recovered values for reward learning rate; r = .71, p < .001, 2-sided. **(b)** Correlation between fitted and recovered values for punishment learning rate; r = .80, p < .001, 2-sided. **(c)** Correlation between fitted and recovered values for action initiation bias; r = .92, p < .001, 2-sided. **(d)** Correlation between fitted and recovered values for temperature parameter; r = .49, p < .001, 2-sided. For presentation purposes, temperature parameter values are log-transformed. This Figure relates to Figure 3c in the main text.

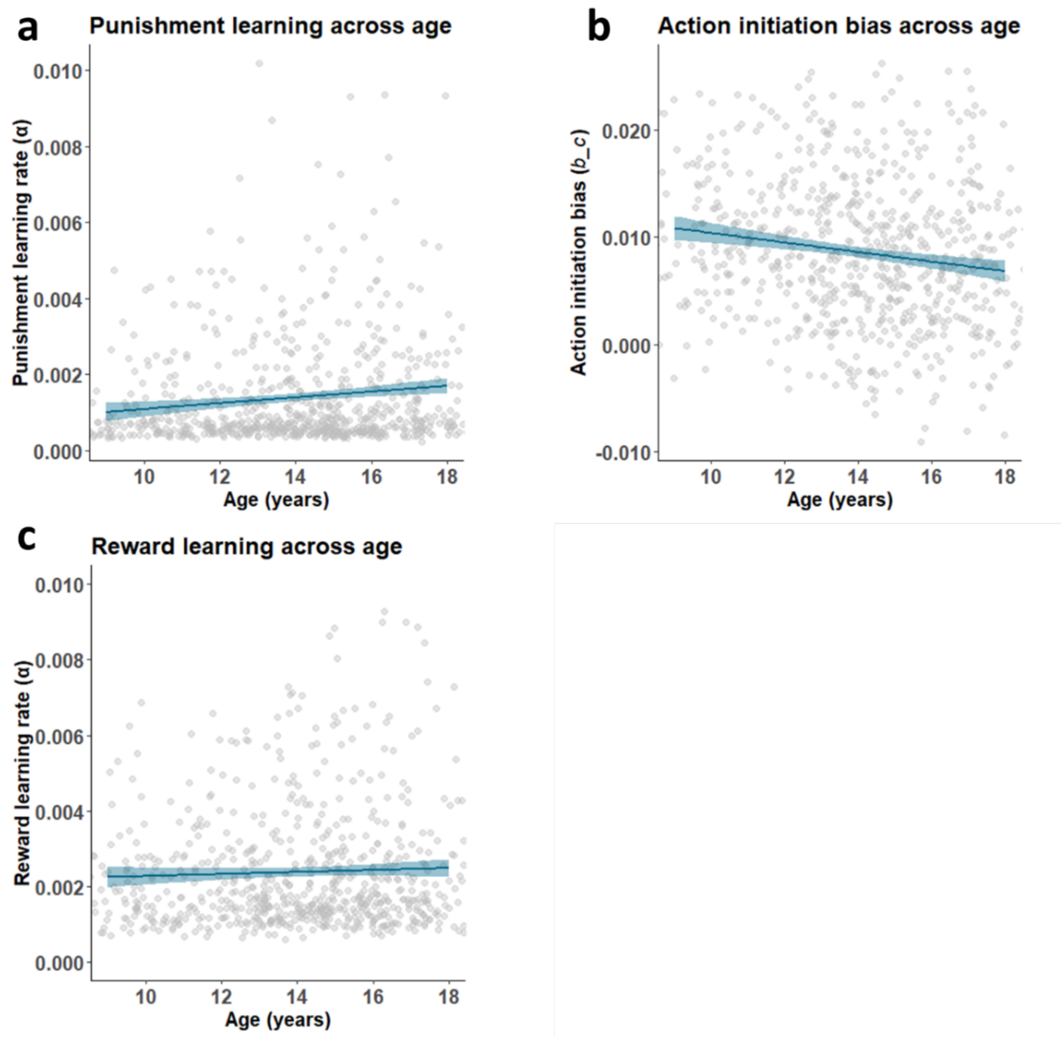

Figure S10. Associations between model parameter values and age as a continuous variable. (a) Associations between punishment learning and age. Punishment learning rates increased with age. **(b)** Associations between action initiation bias and age. Action initiation biases decreased with age. **(c)** Associations between reward learning and age. Reward learning remained stable with age. Error band represents 95% confidence intervals. N = 742.

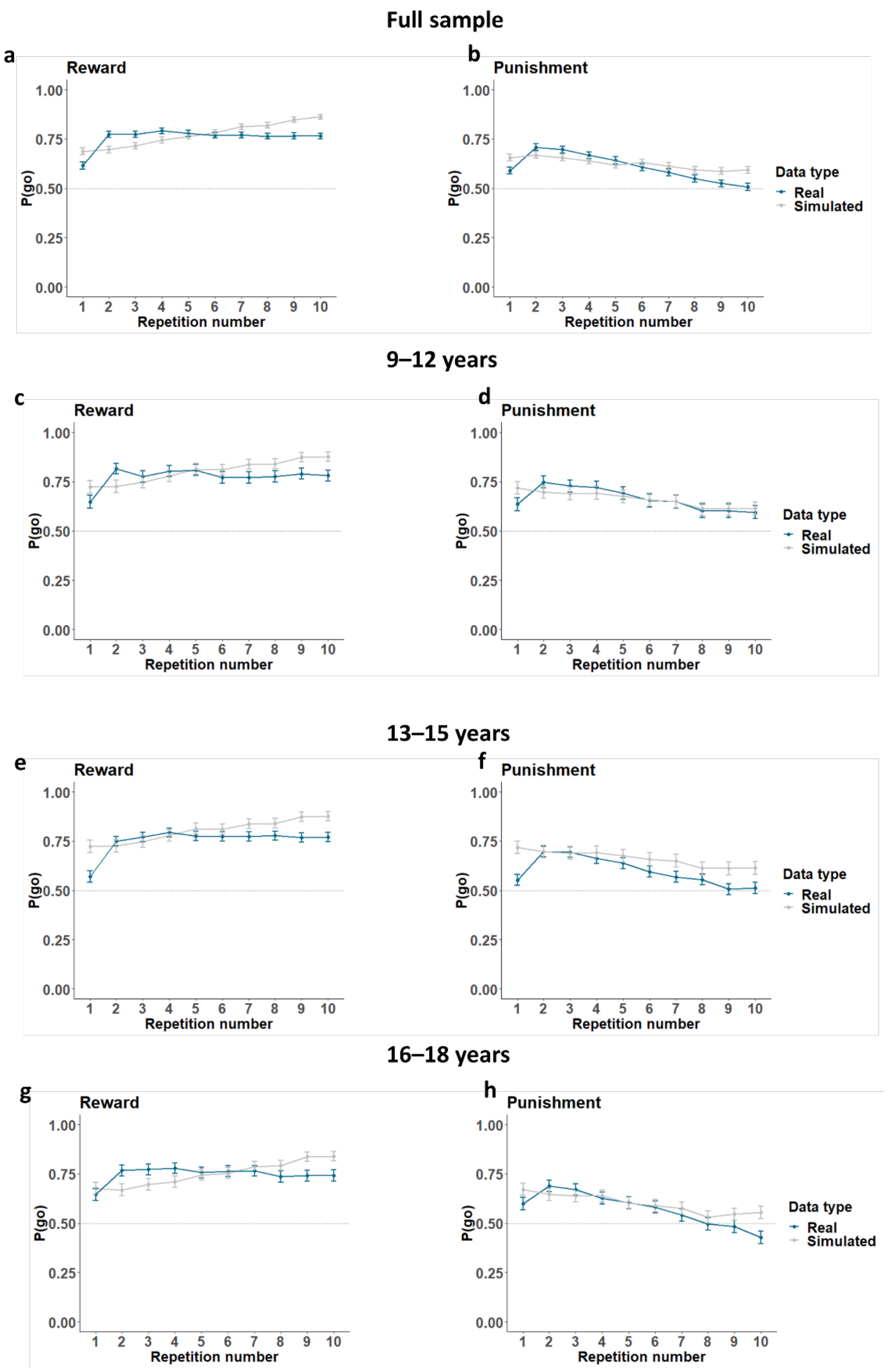

Figure S11. Simulated probability of 'go' response to reward and punishment stimuli across 10 stimulus repetitions. (a) Simulated and real probability of 'go' responses on reward trials, with simulated data generated using the winning model and its median parameter values for the full sample (N = 742). **(b)** Simulated and real probability of 'go' responses on punishment trials for the full sample (N = 742). **(c)-(d)** Simulated and real probability of 'go' responses on reward **(c)** and punishment **(d)** trials for participants aged 9-12 years. **(e)-(f)** Simulated and real probability of 'go' responses on reward **(e)** and punishment **(f)** trials for participants aged 13-15 years.

(g)-(h) Simulated and real probability of 'go' responses on reward **(g)** and punishment **(h)** trials for participants aged 16-18 years. N = 199 aged 9-12, 302 aged 13-15, and 235 aged 16-18.

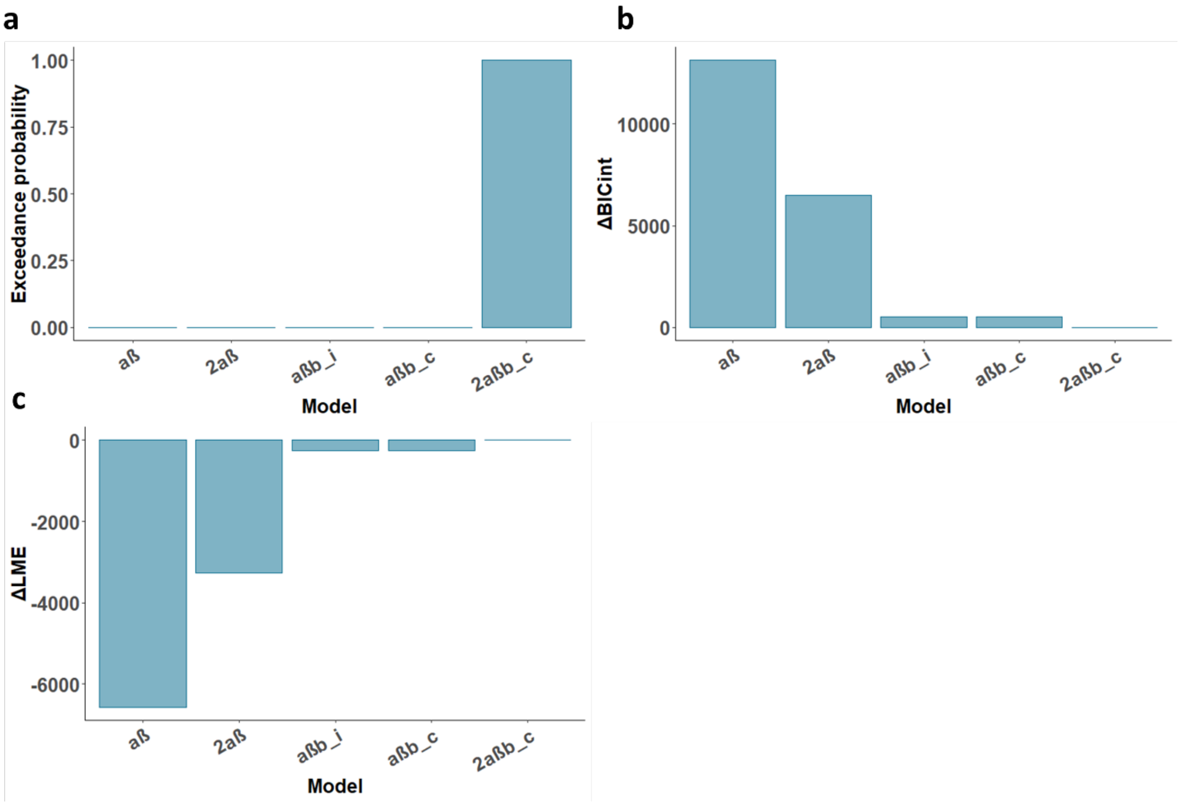

Figure S12. Model comparison with all 832 eligible participants. (a) Exceedance probability for the five computational models that comprised the model space for the larger (N = 832) dataset. The winning model on this measure was the 2αβ*b_c* model. **(b)** ΔBIC_{int}, relative to the winning model (2αβ*b_c*). **(c)** ΔLME, relative to the winning model (2αβ*b_c*).

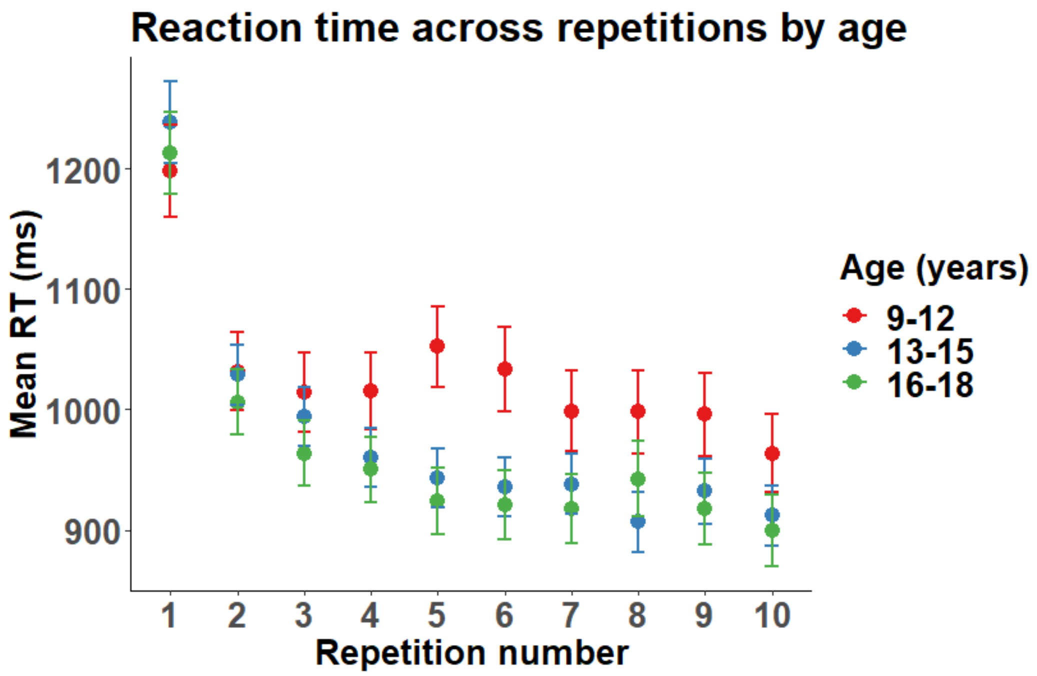

Figure S13. Reaction time for 'go' responses across stimulus repetitions, by age. Reaction times decreased over the course of the task, and this decrease was greater for older participants (linear mixed effects regression: $\beta = -9.69$ [–14.63, –4.75], $t_{(40084)} = -3.85$, $p < .001$, 2-sided). Age was treated as continuous in this analysis; age bins are for plotting only. N = 742.

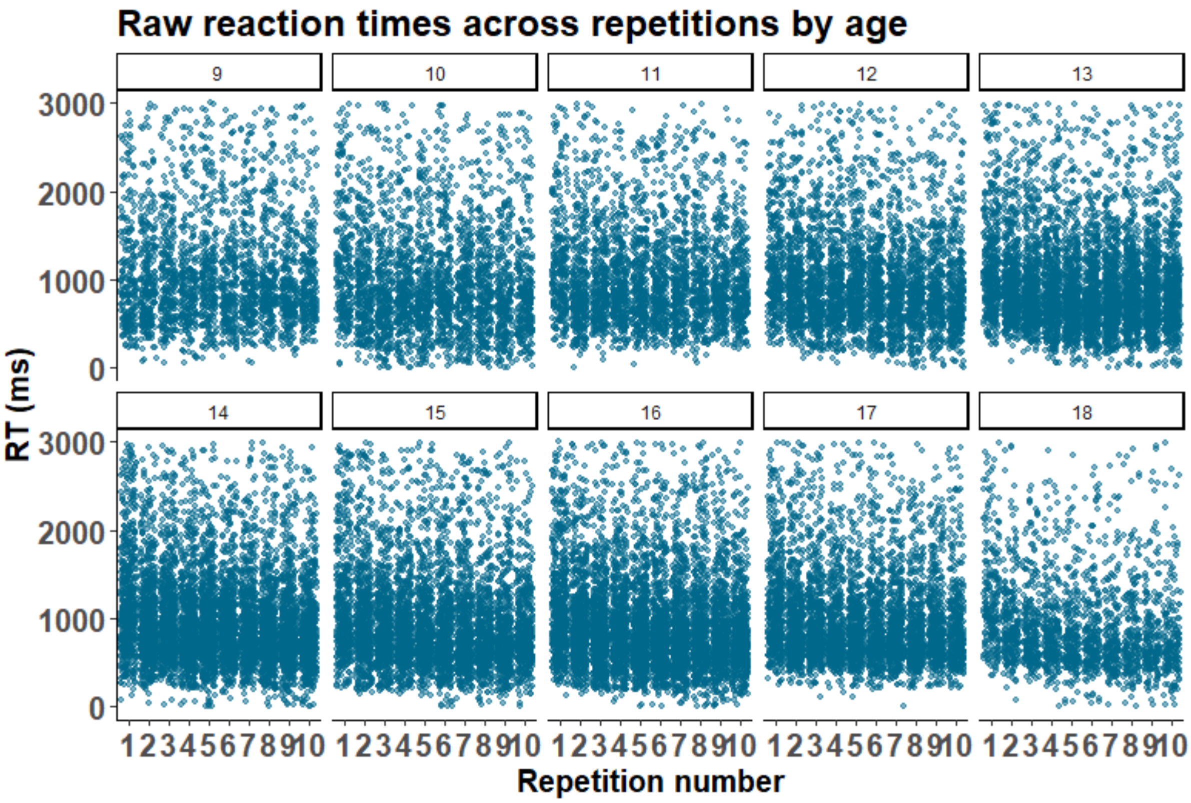

Figure S14. Raw reaction times for 'go' responses across stimulus repetitions, by age. Data points represent raw reaction times for stimulus repetitions 1-10 (x-axis), presented separately by age in years (labelled in panels above each sub-figure). For each stimulus repetition, there are eight trials (one per stimulus) for each of the 742 participants. Participants had up to 3000ms to respond. N = 41 aged 9 years, 48 aged 10, 50 aged 11, 61 aged 12, 86 aged 13, 118 aged 14, 99 aged 15, 112 aged 16, 86 aged 17, and 41 aged 18.

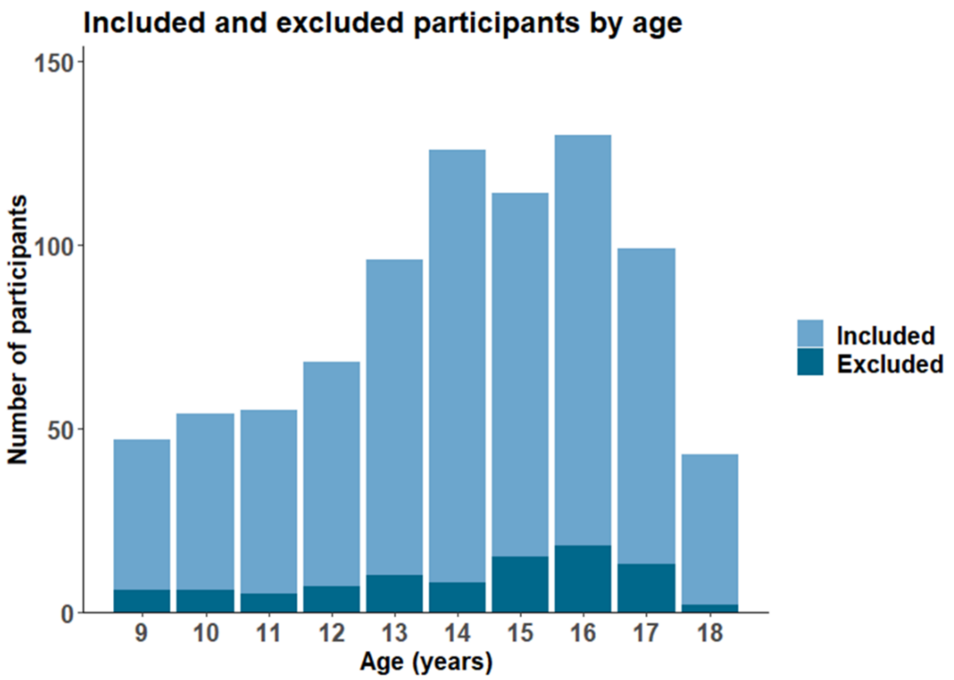

Figure S15. Number of participants included in or excluded from the final sample by age. Exclusions were due to poor data quality. The proportion of exclusions was similar across the age range.

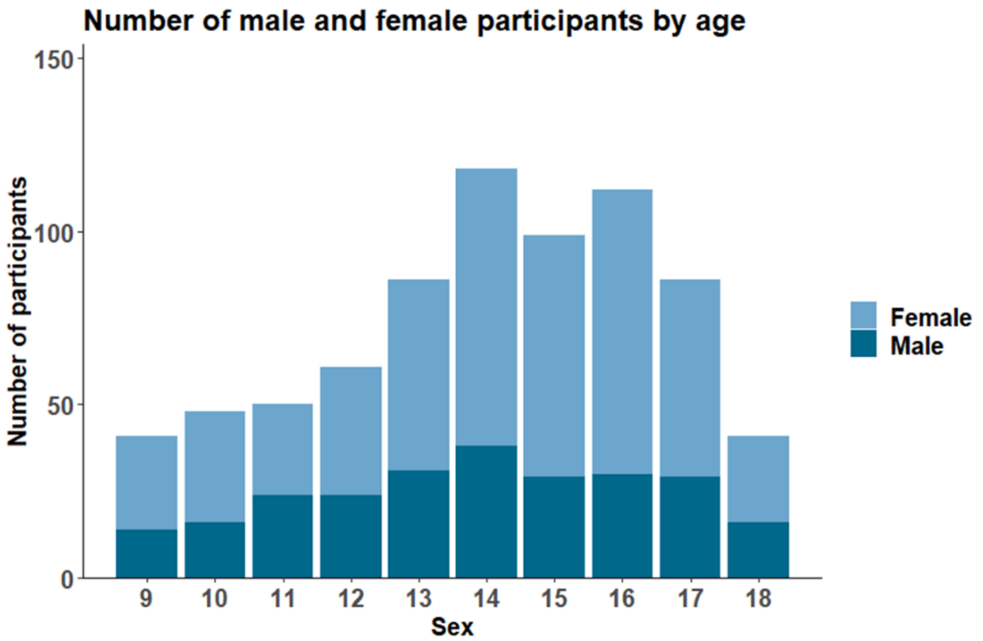

Figure S16. Number of male and female participants by age. The greater proportion of females across the age range reflects a deliberate sampling strategy in the wider dataset.

Supplementary Tables

Table S1. Correlations between demographic variables. Figures are Pearson's *r* [95% confidence intervals]

	**Age**	**Pubertal status**	**Sex**	**IQ**
**Age**	-	0.75<br>[0.72, 0.78]<br>*p* < .001	0.05<br>[–0.02, 0.12]<br>*p* = .20	–0.26<br>[–0.33, –0.2]<br>*p* < .001
**Pubertal status**		-	0.36<br>[0.29, 0.42]<br>*p* < .001	–0.23<br>[–0.29, 0.12]<br>*p* < .001
**Sex**			-	–0.08<br>[–0.00, –0.15]<br>*p* = .04
**IQ**				-

Table S2. Summary of additional analyses

	Main analyses	With model 6	With all eligible participants	With all eligible and outliers	With repetitions 2-10 only
Sample size					
N	742	742	832	832	742
Behavioural analysis: GLMM predicting no. correct responses from age*					
Main effect stimulus repetition	OR = 1.19 [1.17, 1.21], z = 18.56, p < .001)	N/A	OR = 1.17 [1.15, 1.19], z = 19.01, p < .001	N/A	OR = 1.15 [1.13, 1.17], z = 13.87, p < .001
Age*repetition	OR = 1.02 [1.01, 1.04], z = 2.49, p = .01)	N/A	OR = 1.03 [1.01, 1.04], z = 2.97, p = .003	N/A	OR = 1.02 [1.00, 1.04], z = 2.06, p = .04
Main effect age	(OR = 1.08 [1.04, 1.11], z = 4.58, p <.001)	N/A	OR = 1.07 [1.04, 1.10], z = 4.92, p <.001	N/A	OR = 1.08 [1.04, 1.12], z = 4.48, p < .001
Age*repetition* valence	OR = 1.09 [1.05, 1.13], z = 4.65, p <.001)	N/A	OR = 1.10 [1.06, 1.14], z = 5.56, p <.001	N/A	OR = 1.07 [1.03, 1.12], z = 3.32, p < .001
BF_{01} for (no) age difference in reward learning	57.80	N/A	29.11	N/A	112.00
Modelling: model comparison					
Overall winning model	5	N/A (6)	5	5	5
Modelling: GLMMs predicting parameter values from age*					
Main effect of age on punishment learning	β = 0.10 [0.05, 0.15], z = 4.12, p < .001	β = 0.10 [0.05, 0.15], z = 4.26, p < .001	β = 0.11 [0.07, 0.15], z = 4.86, p < .001	β = 0.05 [0.03, 0.07], z = 5.40, p < .001	β = 0.12 [0.07, 0.17], z = 4.59, p < .001
Main effect of age on action initiation bias	β = –0.20 [–0.28, –0.12], z = –4.91, p < .001	β = –0.20 [–0.28, –0.12], z = –4.78, p < .001	β = –0.20 [–0.27, –0.12], z = –5.30, p < .001	β = –0.17 [–0.23, –0.10], z = –5.14, p < .001	β = –0.15 [–0.23, –0.06], z = –3.44, p < .001
Main effect of age on reward learning rate	β = 0.01 [–0.05, 0.07], z = 0.30, p = .77	β = 0.01 [–0.06, 0.07], z = 0.17, p = .86	β = 0.02 [–0.03, 0.08], z = 0.80, p = .42	β = 0.03 [–0.01, 0.07], z = 1.29, p = .20	β = 0.03 [–0.05, 0.11], t = 0.73, p = .46**
BF_{01} for (no) age difference in reward learning rate	13.60	12.62	12.62	11.57	20.00
Main effect of age on temperature parameter	β = 0.002 [–0.07, 0.07], z = 0.08, p = 0.94	β = 0.002 [–0.07, 0.08], z = 0.06, p = 0.95	β = –0.03 [–0.10, 0.03], z = –0.96, p = 0.34	β = –0.03 [–0.10, 0.04], t = –0.85, p = 0.39 **	β = 0.06 [–0.01, 0.14], z = 1.70, p = .09

*All p values reflect 2-sided tests

**These statistics are from a standard linear model without site, due to non-convergence of the mixed (GLMM) model due to model complexity

Table S3. Correlations between model parameters and task performance (proportion of correct responses) for reward and punishment stimuli separately. Figures are Spearman's r [95% confidence intervals] as parameter values were not normally distributed. All tests are 2-sided.

	**β**	**α_r**	**α_p**	***b_c***
**Reward performance**	−0.60<br>[−0.64, −0.54]<br>$p < .001$	0.21<br>[0.13, 0.28]<br>$p < .001$	−0.01<br>[−0.09, 0.06]<br>$p = .72$	0.82<br>[0.79, 0.84]<br>$p < .001$
**Punishment performance**	0.11<br>[0.03, 0.18]<br>$p < .001$	0.19<br>[0.11, 0.26]<br>$p < .001$	0.57<br>[0.52, 0.62]<br>$p < .001$	−0.86<br>[−0.88, −0.84]<br>$p < .001$

Notes: β: temperature parameter, α_r: reward learning rate, α_p: punishment learning rate, *b_c*: constant action initiation bias.

Table S4. Correlations between model parameters (Spearman's *r* [95% confidence intervals]). All tests are 2-sided.

	**β**	**α_r**	**α_p**	***b_c***
**β**	-	−0.58<br>[−0.63, −0.53]<br>$p < .001$	−0.21<br>[−0.28, −0.14]<br>$p < .001$	−0.36<br>[−0.42, −0.30]<br>$p < .001$
**α_r**		-	−0.10<br>[−0.17, −0.03]<br>$p = .01$	−0.18<br>[−0.25, −0.11<br>$p < .001$
**α_p**			-	−0.19<br>[−0.26, −0.12]<br>$p < .001$
b_c				-

Notes: β: temperature parameter, α_r: reward learning rate, α_p: punishment learning rate, *b_c*: constant action initiation bias.

Supplementary References

1. Kahneman D, Tversky A. Prospect Theory: An Analysis of Decision under Risk. *Econometrica*. 1979;47(2):263-291. doi:10.2307/1914185

2. Yang K, Qi H. The Nonlinear Impact of Task Rewards and Duration on Solvers' Participation Behavior: A Study on Online Crowdsourcing Platform. *J Theor Appl Electron Commer Res*. 2021;16(4):709-726. doi:10.3390/jtaer16040041

3. DiPalantino D, Vojnovic M. Crowdsourcing and all-pay auctions. In: *Proceedings of the 10th ACM Conference on Electronic Commerce*. EC '09. Association for Computing Machinery; 2009:119-128. doi:10.1145/1566374.1566392

4. Nussenbaum K, Hartley CA. Reinforcement learning across development: What insights can we draw from a decade of research? *Dev Cogn Neurosci*. 2019;40:100733. doi:10.1016/j.dcn.2019.100733

5. Williams BR, Ponesse JS, Schachar RJ, Logan GD, Tannock R. Development of inhibitory control across the life span. *Dev Psychol*. 1999;35(1):205-213. doi:10.1037/0012-1649.35.1.205

6. Kaufman J, Birmaher B, Brent D, et al. Schedule for Affective Disorders and Schizophrenia for School-Age Children-Present and Lifetime Version (K-SADS-PL): Initial Reliability and Validity Data. *J Am Acad Child Adolesc Psychiatry*. 1997;36(7):980-988. doi:10.1097/00004583-199707000-00021

7. American Psychiatric Association. *Diagnostic and Statistical Manual of Mental Disorders (DSM-5)*. American Psychiatric Association; 2013.

8. Eekhout I, de Vet, H.C., Twisk, J.W., Brand, J.P., de Boer, M.R., Heymans, M.W. Missing data in a multi-item instrument were best handled by multiple imputation at the item score level. *J Clin Epidemiol*. 2014;67(3):335-342. doi:https://doi.org/10.1016/j.jclinepi.2013.09.009

9. Liu, Y., De, A. Multiple imputation by fully conditional specification for dealing with missing data in a large epidemiologic study. *Int J Stat Med Res*. 2015;4(3):287. doi:doi: 10.6000/1929-6029.2015.04.03.7
