# Supplementary material for: Action initiation and punishment learning differ from childhood to adolescence while reward learning remains stable
Source: Nat Commun. 2023 Sep 14;14:5689. doi: 10.1038/s41467-023-41124-w (PMC10502052; doi:10.1038/s41467-023-41124-w)
Supplement: Supplementary file 3 — Reporting Summary [file 41467_2023_41124_MOESM3_ESM.pdf]

nature portfolio

Corresponding author(s): Ruth Pauli & Patricia Lockwood

Last updated by author(s): Jul 5, 2023

Reporting Summary

Nature Portfolio wishes to improve the reproducibility of the work that we publish. This form provides structure for consistency and transparency in reporting. For further information on Nature Portfolio policies, see our Editorial Policies and the Editorial Policy Checklist.

Statistics

For all statistical analyses, confirm that the following items are present in the figure legend, table legend, main text, or Methods section.

n/a	Confirmed	
☐	☒	The exact sample size (n) for each experimental group/condition, given as a discrete number and unit of measurement
☐	☒	A statement on whether measurements were taken from distinct samples or whether the same sample was measured repeatedly
☐	☒	The statistical test(s) used AND whether they are one- or two-sided<br>*Only common tests should be described solely by name; describe more complex techniques in the Methods section.*
☐	☒	A description of all covariates tested
☐	☒	A description of any assumptions or corrections, such as tests of normality and adjustment for multiple comparisons
☐	☒	A full description of the statistical parameters including central tendency (e.g. means) or other basic estimates (e.g. regression coefficient) AND variation (e.g. standard deviation) or associated estimates of uncertainty (e.g. confidence intervals)
☐	☒	For null hypothesis testing, the test statistic (e.g. F, t, r) with confidence intervals, effect sizes, degrees of freedom and P value noted<br>*Give P values as exact values whenever suitable.*
☐	☒	For Bayesian analysis, information on the choice of priors and Markov chain Monte Carlo settings
☒	☐	For hierarchical and complex designs, identification of the appropriate level for tests and full reporting of outcomes
☐	☒	Estimates of effect sizes (e.g. Cohen's d, Pearson's r), indicating how they were calculated

Our web collection on statistics for biologists contains articles on many of the points above.

Software and code

Policy information about availability of computer code

Data collection	Learning task data were collected in E-Prime 1.0.
Data analysis	Analyses were conducted in Matlab (R2019b) and R (4.1.1. and 4.1.2). Data and code are available at https://osf.io/d2zp4/

For manuscripts utilizing custom algorithms or software that are central to the research but not yet described in published literature, software must be made available to editors and reviewers. We strongly encourage code deposition in a community repository (e.g. GitHub). See the Nature Portfolio guidelines for submitting code & software for further information.

Data

Policy information about availability of data

All manuscripts must include a data availability statement. This statement should provide the following information, where applicable:

- Accession codes, unique identifiers, or web links for publicly available datasets
- A description of any restrictions on data availability
- For clinical datasets or third party data, please ensure that the statement adheres to our policy

The data included in this study have been deposited in the OSF repository and are available at https://osf.io/d2zp4/.

Research involving human participants, their data, or biological material

Policy information about studies with human participants or human data. See also policy information about sex, gender (identity/presentation), and sexual orientation and race, ethnicity and racism.

Reporting on sex and gender	We use the term 'sex' to refer to biological sex. Sex was used as a covariate of no interest in our main analyses but report key statistics around sex, such as the proportion of males and females in the whole sample and across the age range.
Reporting on race, ethnicity, or other socially relevant groupings	We did not collect data on race or ethnicity. We controlled for site of data collection in our statistical models. We estimated participants' socioeconomic status (SES) based on estimated household income and used this as a covariate of no interest in our statistical models.
Population characteristics	We collected data on age, pubertal status, sex, SES, and site of data collection. During screening, we checked that all participants were free from current psychopathology. As there were some associations between age and these measures (see Table S1), we included sex and IQ as covariates of no interest in all analyses of participants' behavioural responses and model parameters.
Recruitment	Please see below.
Ethics oversight	The FemNAT-CD project received ethical approval from the relevant local ethics committees, as follows: Aachen: Ethik Kommission Medizinische Fakultät der Rheinisch Westfälischen Technischen Hochschule Aachen (EK027/14). Amsterdam: Medisch Etische Toetsingscommissie (2014.188). Athens: Election Committee of the First Department of Psychiatry, Eginition University Hospital (641/9.11.2015). Barcelona: Child and Adolescent Mental Health—University Hospital Mutua Terrassa (acta 12/13). Basel: Ethik Kommission Nordwest- und Zentralschweiz (EKNZ 336/13). Bilbao: Hospital del Basurto. Birmingham and Southampton: University Ethics Committee and National Health Service Research Ethics Committee (NRES Committee West Midlands, Edgbaston; REC reference 3/WM/0483). Dublin: SJH/AMNCH Research Ethics Committee (2014/04/Chairman (3)). Frankfurt: Ethik Kommission Medizinische Fakultät Goethe Universität Frankfurt am Main (445/13). Szeged (Hungary): Egészségügyi Tudományos Tanács Humán Reprodukciós Bizottság (CSR/039/00392–3/2014). This study was conducted in accordance with the ethical standards of the 1964 Declaration of Helsinki and its later amendments.

Note that full information on the approval of the study protocol must also be provided in the manuscript.

Field-specific reporting

Please select the one below that is the best fit for your research. If you are not sure, read the appropriate sections before making your selection.

☐ Life sciences ☒ Behavioural & social sciences ☐ Ecological, evolutionary & environmental sciences

For a reference copy of the document with all sections, see nature.com/documents/nr-reporting-summary-flat.pdf

Behavioural & social sciences study design

All studies must disclose on these points even when the disclosure is negative.

Study description	We analysed quantitative data derived from participants' behavioural responses on a learning task and derived model parameters, plus covariates such as IQ, age, and sex.
Research sample	The sample consisted of 742 children and adolescents aged 9-18 years (491 girls), recruited from 11 European countries. All participants were free from psychiatric, behavioural, or developmental disorders, and were of normal intelligence. Participants were recruited from schools and community sources such as youth clubs, as well as by word of mouth and responses to study advertisements. The research sample was chosen based on convenience sampling and not explicitly to ensure representativeness of the populations in the respective countries.
Sampling strategy	Participants were selected from the FemNAT-CD study database (see recruitment details above). We selected all participants who were free from psychiatric, behavioural, and developmental disorders, and who had available data for the learning task. We used convenience sampling, ensuring that recruited participants were free from our exclusion criteria.
Data collection	Participants completed the task on a laptop, with a researcher present in the room to provide guidance if necessary. All researchers involved in data collection were blind to the study hypotheses.
Timing	Data collection ran from 2014 to 2018.
Data exclusions	Eight hundred and thirty-nine participants were eligible for inclusion. We screened the data to exclude participants with poor task performance. Five participants never responded, four responded to every trial, six scored below zero points on the task (indicating deliberate punishment-seeking and reward-avoidance), and 96 responded to fewer than half of the reward trials (i.e., trials where responding was the correct behaviour).
Non-participation	N/A - these data were collected as part of a previous consortium study. (Information on specific dropouts for the current subset of data are not available in the consortium data).

Randomization	Relevant variables (e.g., IQ, sex) were entered as covariates into regression models.

Reporting for specific materials, systems and methods

We require information from authors about some types of materials, experimental systems and methods used in many studies. Here, indicate whether each material, system or method listed is relevant to your study. If you are not sure if a list item applies to your research, read the appropriate section before selecting a response.

Materials & experimental systems

n/a	Involved in the study	
☒	☐	Antibodies
☒	☐	Eukaryotic cell lines
☒	☐	Palaeontology and archaeology
☒	☐	Animals and other organisms
☒	☐	Clinical data
☒	☐	Dual use research of concern
☒	☐	Plants

Methods

n/a	Involved in the study	
☒	☐	ChIP-seq
☒	☐	Flow cytometry
☒	☐	MRI-based neuroimaging
